# Infectious Diseases in Private Houses

**Published:** 1892-06-11

**Authors:** Walter Maguire


### Editor's Letter Box.

[Our correspondents are reminded that prolixity of statement is a great bar to publication, and that brevity of style and conciseness of statement greatly facilitate early insertion.]

### INFECTIOUS DISEASES IN PRIVATE HOUSES.

Mr. Walter Maguire writes: A deputation of ratepayers of Marylebone waited upon the Marylebone Vestry, last Thursday, to present a petition signed by 500 of the principal tradesmen and others in the district protesting against two houses in Weymouth Street being used for reception of patients suffering from fever and other infectious diseases. I understand many of these cases were brought from a school in Westminster in ambulances belonging to the Metropolitan Asylums District Board. Now, sir, is it usual for public property to be used for private purposes in this manner, as I have always thought that these ambulances were intended for the removal of patients from private residences to one or other of the Metropolitan Asylums District Board's hospitals? Did the Board sanction the use of these ambulances for this purpose, and if so, by what right? Again, why should we have infected cases thrown into a thickly populated parish like Marylebone, to the purification of other parishes and to the danger of the residents here, and the depreciation of their property and means of livelihood? Surely the Local Government Board had better wake up. With apologies for troubling you.

